# Health-related quality of life in young Norwegian survivors of out-of-hospital cardiac arrest related to pre-arrest exercise habits

**DOI:** 10.1016/j.resplu.2023.100478

**Published:** 2023-10-05

**Authors:** Cecilie Benedicte Isern, Birgitta Blakstad Nilsson, Andrew Garratt, Jo Kramer-Johansen, Ingvild B.M. Tjelmeland, Hilde Moseby Berge

**Affiliations:** a Division of Prehospital Services, Oslo University Hospital, Ullevål Hospital, P.O. Box 4956 Nydalen, NO-0424 Oslo, Norway; b Faculty of Medicine, Institute of Clinical Medicine, University of Oslo, P.O. Box 1078 Blindern, NO-0316 Oslo, Norway; c Oslo Sports Trauma Research Center, Department of Sports Medicine, Norwegian School of Sport Sciences, P.O. Box 4014 Ullevål Stadion, NO-0806 Oslo, Norway; d Section for Physiotherapy, Department of Clinical Services, Division of Medicine, Oslo University Hospital, Oslo, Norway; e Department of Rehabilitation Science and Health Technology, Faculty of Health Sciences, OsloMet – Oslo Metropolitan University, Oslo, Norway; f Norwegian Institute of Public Health, Oslo, Norway, Health Services Research Centre, Akershus University Hospital, Lorenskog, Norway; g University Hospital Schleswig-Holstein, Institute for Emergency Medicine, Holzkoppelweg 8-12, Kiel, Germany; h Department of General Practice, Institute of Health and Society, University of Oslo, P.O. Box 1130 Blindern, NO-0318 Oslo, Norway

**Keywords:** Cardiac arrest, Population based study, Long-term outcome, Patient reported outcome, SF-36, Young age, Exercise

## Abstract

**Aim:**

To compare health-related quality of life (HRQoL) in young survivors of out-of-hospital cardiac arrest (OHCA) in Norway with an age and sex-matched reference population and to assess the associations between exercise volume prior to OHCA and HRQoL after.

**Methods:**

We present data from survivors aged 18–50 years registered with OHCA in the Norwegian Cardiac Arrest Registry between January 1st 2015 and December 31st 2017. Survivors were invited to answer two questionnaires; (1) the Short Form 36 (SF-36) Health Survey Version 1, and (2) about exercise habits prior to OHCA. Respondents were randomly matched 1:1 for age and sex with a reference population (data were available from the Norwegian Centre for Research Data).

**Results:**

Of the 175 survivors invited, 95 (54%) responded, median age was 44 (range 35–48) years, 26 (27%) females. Valid results for SF-36 were available for 91 survivors, of whom 87 reported pre-OHCA exercise-volume. Prior to OHCA, 21 did no regular exercise, 44 exercised 1–4 hours/week and 22 exercised ≥5 hours/week. Compared to the reference population survivors had significantly (*p* < 0.01) poorer SF-36 scores for scales relating to physical- and mental health. SF-36 scale scores were similar in survivors who did and did not exercise regularly. Within the regular exercisers, survivors reporting ≥5 hours of exercise/week had better SF-36 scores than those exercising less.

**Conclusion:**

Poorer HRQoL in survivors compared to the reference population should prompt us to explore how treatment and rehabilitation could be improved and adapted. More exercise before OHCA favoured better HRQoL after, which aligns well with the recognised positive association between HRQoL and physical activity in general.

## Introduction

Young adults make up a small proportion of those who experience and survive out-of-hospital cardiac arrest (OHCA) but constitute an important group as they have potentially many remaining years of life.^1^ To improve their long-term outcome beyond survival, motivation and prioritisation should be guided by knowledge of how young survivors experience their life.

Health-related quality of life (HRQoL) is an important measure of the patients’ perspective of long-term outcomes in survivors of OHCA,^2^ but we are only aware of one study that specifically assessed HRQoL in young adult survivors.^3^ Many patient-reported outcome measures are available for assessing HRQoL. Patient-reported outcome measures are not straightforward, and lack of standardisation hinders comparisons across studies which might also differ in terms of inclusion criteria.^4^, ^5^ In a recent qualitative study, young adult survivors reported physical and cognitive sequelae along with emotional instability.^6^ Furthermore, age-specific challenges with returning to everyday life including responsibility for children and school or work commitments were important concerns. Regular physical activity is a modifiable variable positively associated with HRQoL in the general population.^7^, ^8^ It is plausible that exercise habits prior to OHCA could be an important contributory factor to post-OHCA HRQoL outcomes among young adults, but no one has investigated this.

The aims of the present study were to compare HRQoL in young survivors of OHCA in Norway with an age- and sex-matched reference population and to assess the association between exercise volume prior to the OHCA and HRQoL after OHCA.

## Methods

### Study design and population

**Fig. 1:**
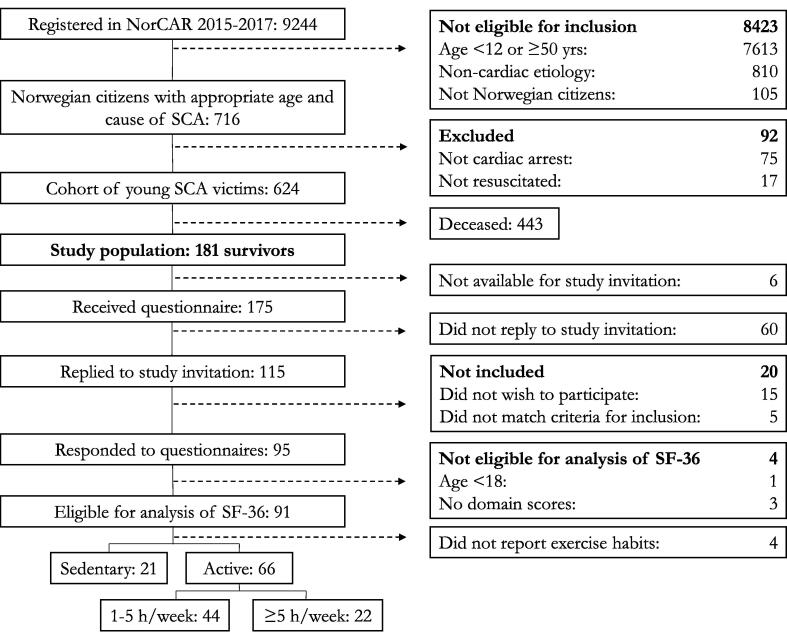
Flow chart of inclusion. Starting with available registrations in the Norwegian Cardiac Arrest Registry, continuing with patients eligible for inclusion in the main project describing young victims of OHCA in Norway (*n* = 624): Survivors eligible for inclusion in this study (*n* = 181 survivors), respondents to the questionnaire (*n* = 95); survivors eligible for analysis of SF-36 (*n* = 91) and presented by exercise-habits prior to OHCA.

This cross-sectional study included survivors of OHCA,^2^ registered in the Norwegian Cardiac Arrest Registry (NorCAR) between 1st of January 2015 and 31st of December 2017.^9^ This study population originates from a larger study population of young Norwegian victims of OHCA with presumed cause registered as cardiac, drowning or respiratory failure, aged between 12 and 50 years at time of the OHCA.^10^ Patients without a national identity number were excluded. SF-36 is only validated for persons ≥18 years of age, and data on HRQoL for survivors <18 years of age were therefore excluded before analysis. Fig. 1 shows the study flow chart.

### Data collection and definitions

**Fig. 2:**
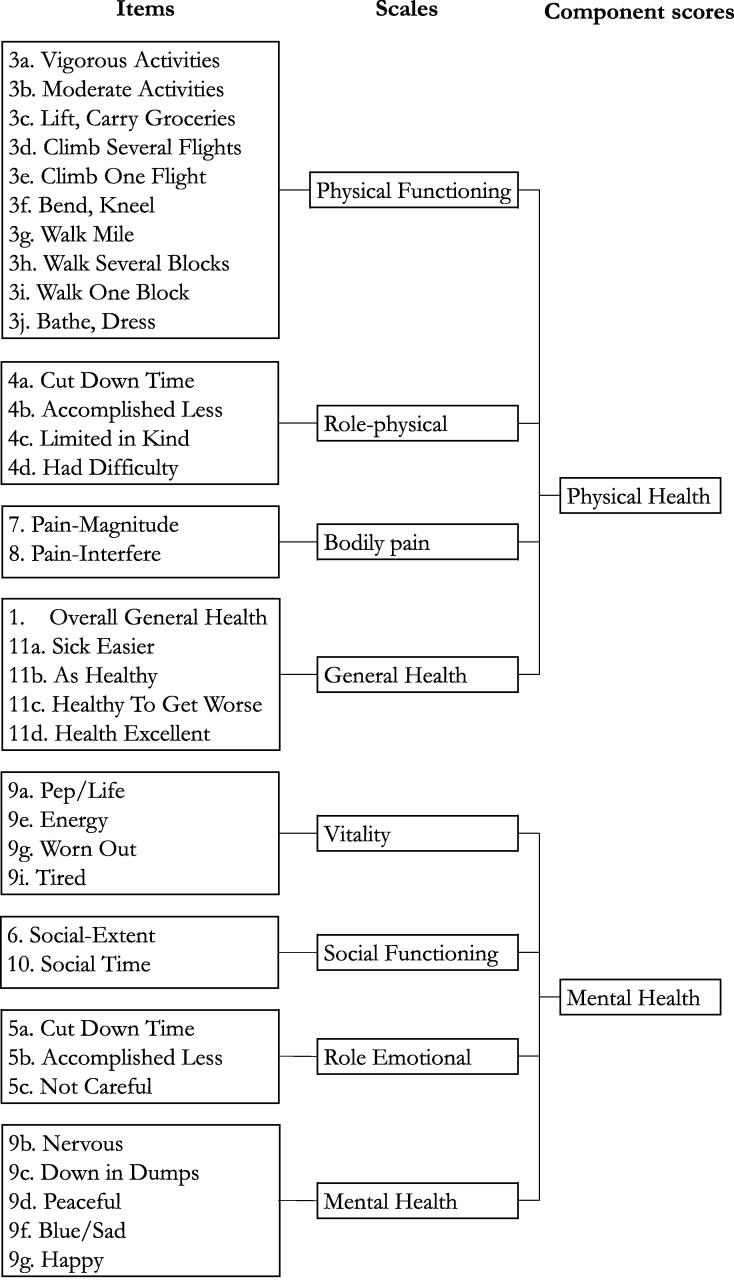
Overview of how the SF-36-items and their contribution to 8 scale and 2 component scores. The figure is adapted from Ware et al.^45^

Characteristics about the OHCA event are collected from NorCAR. Survivors eligible for inclusion received a paper-based study invitation, and two questionnaires, between one and three years after the OHCA. Non-respondents received one reminder. The first questionnaire included the Short Form 36 (SF-36) Health Survey Version 1.^11^ Responses to the 36 items contribute to eight health scales (Fig. 2). The resulting scale scores range from 0 (worst possible health state) to 100 (best possible health state) and are computed if there are responses to half or more contributing items. Based on weights derived from factor analysis, these scale scores contribute to physical and mental component summary scores, which are standardised to have a mean of 50 and standard deviation of 10 (Fig. 2).^11^, ^12^ The reliability, validity and responsiveness of the SF-36 are well documented in general populations and in patients with heart disease.^11^, ^13^

This paper is part of a PhD-project with the overall aim of contributing to the knowledge base for the young Norwegian OHCA population, both in general and in relation to exercise. The first and last author designed the second questionnaire to collect information corresponding to the recommendations from the Sport Cardiology Section of the European Association for Cardiovascular Prevention and Rehabilitation for pre-participation cardiovascular screening of athletes.^14^ The questionnaire includes 34 pre-defined response alternatives and 22 free-text questions (Supplemental 1). As reported here, it included information about exercise habits (exercise volume and type of exercise conducted), athletic level and exercise-related OHCA. Prior to the study, the questionnaire was tested in four different user representatives comprising an adult survivor, and next-of-kin to a surviving child, a deceased child, and a deceased adult. Following their feedback, the introductory text to some questions was changed to improve understanding.

The reference population was based on a cross-sectional postal survey of members of the general population representative for Norway conducted as part of “Level of Living Survey 2002”.^15^ The questionnaire included the SF-36 mailed in the period 15th November 2002 to 15th May 2003. This data was available from the Norwegian Centre for Research Data. A total of 3271 participants were eligible for matching. SF-36 data were found to have adequate measurement properties for use as reference population data in Norwegian studies.^11^

The respondents were asked to report exercise volume as the average hours of exercise per week the last year prior to OHCA. In the questionnaire, exercise was defined as physical strain that would lead to sweat or increased heart rate. For the purpose of analysis, we grouped the population in those who did no regular exercise versus those who reported regular exercise prior to OHCA, the latter will be referred to as regular exercisers. Within the group of regular exercisers, we did sub-analysis according to exercise volume of < or ≥5 hours/week. The term exercise habits include type of exercise in addition to exercise volume. Exercise-related OHCA was defined as OHCA during or within 60 minutes after exercise.^2^ Athletic level was self-reported among regular exercisers as recreational or competitive.

With the perspective that all study participants were younger than the median age of other OHCA populations, we defined them as “young OHCA survivors”.^9^, ^16^, ^17^ To evaluate outcome among survivors, NorCAR registers a Cerebral Performance Category (CPC) based on information from the medical discharge report. The CPC scores range from good cerebral performance (1) to brain death (5).^18^

### Statistical analysis

Each patient in the study population was randomly matched 1:1 for age and sex with a control from the reference population. SF-36 scale scores are presented as mean and standard deviation (SD) for comparability with other studies.^19^, ^20^ For continuous variables, mean (SD) and median with interquartile range (IQR) were used as appropriate. For normally distributed variables, we used student t-test to compare means, otherwise, we used the Mann-Whitney U test. We compared categorical variables with the Chi-square test, and if one or more cells had an expected value of less than five, we used Fisher's Exact Test. Multiple testing for the SF-36 meant that *p*-values <0.01 were regarded as statistically significant.

Mean difference between the groups was presented with 95% confidence interval (95% CI). We used the minimal important change criteria reported for SF-36 version 2, to evaluate the clinical importance of the between-group differences for the SF-36 scale scores collected from our study population by SF-36 version 1 (Table 2, Table 3).^21^ SF-36 version 2 has different response scales for the two roles-limitations domains. Using the same ratio of minimal to state change ^22^, the estimated minimal important change for the role-physical was 37.7, and 33.33 for the role-emotional scale. For the physical- and mental component summary scores, we recognised changes of ≥4 as a minimal important change.^13^, ^23^, ^24^

**Table 1:** Demographic and event characteristics for young Norwegian survivors of OHCA with presumed cardiac cause, grouped by self-reported exercise habits prior to the OHCA.

	Overall					Regular exercisers				
	No regular exercise		Regular exercisers			1–4 h of exercise/week		≥5h of exercise/week		
	*n*	%	*n*	%	*P*-value	*n*	%	*n*	%	*P*-value
Overall	21		66			44		22		
Age, median (IQR)	48 (44–50)		45 (33–50)		0.07	44 (36–50)		45 (23–49)		0.41
Gender, female	6	29	20	30	0.88	12	27	8 (36)	36	0.45
OHCA, not witnessed by EMS	18		53			35		18		
Witnessed by bystander	17	94	51	96	1.00	33	94	18	100	0.54
Bystander CPR^a^	16	94	47	94	1.00	31	94	16	94	1.00
First rhythm shockable†^,b^	19	95	51	88	0.67	33	89	18	86	0.70
Exercise-related SCA	0	0	20	30	0.002	12	27	8	36	0.45
CPC = 1^c^	21	100	42	76	0.015	24	69	18	90	0.10
Athletic level^d^										
Recreational	–	–	50	83	–	37	95	13	62	0.002
Competitive	–	–	10	17	–	2	5	8	38	0.002

Numbers are presented as *n* (%), except for age.

Missing cases are excluded from the denominator, resulting in (n – missing) cases per respective groups (Groups: No regular exercise/Regular exercisers/“1–5 hours of exercise/week”/“≥5 hours of exercise/week”); missing: ^a^1/3/2/1, ^b^1/8/7/1, ^c^0/11/9/2,^d^–/0/5/1.

Abbreviations: SCA (Sudden Cardiac Arrest), EMS (Emergency Services), CPC; Cerebral Performance Category.

† The nominator include cases that were successfully defibrillated before arrival of EMS, cases that for other reasons had circulation at arrival of EMS are counted as missing.

**Table 2:** Mean (SD) SF-36 scale- and component summary scores for the study population (*n* = 91) of young Norwegian survivors of OHCA with presumed cardiac cause, compared to an age- and sex-matched Norwegian reference population.

	Study population		Norwegian reference population					
	Mean	SD	Mean	SD	*P*-value	Mean difference	95% CI	Minimal important change
Domain scores								
Physical function	84.3	21.9	93.0	13.0	0.001	8.8	3.5–14.1	*7,5*
Role physical	70.1	43.2	83.0	33.1	0.09	12.9	1.7–24.2	*33,33*
Bodily pain	74.4	28,3	80.5	22.4	0.22	6.1	−1.4–13.5	*10*
General health	63.7	25.1	75.6	19.9	0.001	11.9	5.3–18.5	*7,5*
Vitality	56.0	28.2	64.6	19.8	0.09	8.6	1.5–15.7	*9,38*
Social function	75.3	29.4	90.1	16.0	<0.001	14.8	1.5–15.7	*12,5*
Role emotional	73.6	39.6	87.9	28.8	0.003	14.3	4.1–24.4	*37,7*
Mental health	74.1	22.7	82.8	13.6	0.02	8.8	3.3–14.2	*7,5*
Summary scores								
Physical component	48.4	12.2	53.3	8.2	0.007	4.9	1.9–8.0	*4*
Mental component	48.1	12.5	53.7	8.0	0.004	5.6	2.5–8.7	*4*

SF-36 domains are scored from 0-100 where 0 is the worst and 100 is the best possible health. The summary scores; physical- and mental component scores, are scored to have a mean of 50 and a standard deviation of 10 in the US general population. Abbreviations: CI; Confidence Interval and SD; Standard Deviation.

**Table 3:** Mean (SD) SF-36 scale- and summary scores for the study population (*n* = 91) of young Norwegian survivors of OHCA with presumed cardiac cause grouped by self-reported exercise habits prior to the OHCA.

	Overall							Regular exercisers							
	No regular exercise		Regular exercisers					<5h of exercise/ week		≥5h of exercise/ week					
Patients, *n*	21		66					44		22					
	Mean	SD	Mean	SD	*P*-value	Mean difference	95% CI	Mean	SD	Mean	SD	*P*-value	Mean difference	95% CI	Minimal important change
Domain scores															
Physical function	82.3	24.0	84.6	21.9	0.65	2.3	−8.9–13.4	82.2	22.5	89.3	20.3	0.08	7.1	−4.2–18.5	*7,5*
Role physical	64.3	44.4	71.6	43.6	0.54	7.3	−14.5–29.1	65.9	46.4	83.0	35.7	0.16	17.0	−5.5–39.6	*33,33*
Bodily pain	68.2	30.8	76.6	28.0	0.21	8.1	−6.2–22.3	73.0	29.5	83.8	23.5	0.16	10.9	−3.7–25.5	*10*
General health	60.2	25.0	63.7	25.5	0.51	3.5	−9.2–16.1	57.7	25.1	75.7	22.3	0.004	18.0	5.3–30.6	*7,5*
Vitality	55.2	30.8	55.6	28.2	0.99	2.0	−12.3–16.3	50.6	25.3	65.4	31.5	0.002	20.9	7.0–34.9	*9,38*
Social function	75.6	31.5	74.0	29.4	0.61	1.5	−16.4–13.3	71.0	30.7	80.1	26.1	0.26	9.1	−6.2–24.4	*12,5*
Role emotional	69.8	44.6	73.2	38.9	0.995	3.4	−16.7–23.5	69.7	41.2	80.3	33.6	0.36	10.6	−9.7–30.9	*37,7*
Mental health	74.9	24.1	73.4	22.9	0.53	0.4	−11.3–10.5	71.7	21.0	76.7	26.5	0.03	8.7	−2.4–19.7	*7,5*
Summary scores															
Physical component	46.7	12.8	48.7	12.4	0.39	2.0	−4.2–8.2	46.6	12.5	53.2	11.1	0.0098	6.6	0.2–13.0	*4*
Mental component	47.3	13.3	48.0	12.6	0.95	0.6	−6.1–7.4	45.7	12.5	52.7	11.8	0.009	6.9	0.5–13.4	*4*

SF-36 domains are scored from 0-100 where 0 is the worst and 100 is the best possible health. The summary scores; physical- and mental component summary scores, are scored to have a mean of 50 and a standard deviation of 10 in the US general population. Abbreviations: CI; Confidence Interval and SD; Standard Deviation.

Statistical analysis was performed in statistical software package (IBM SPSS Statistics for Windows Corp. Released 2019, Version 26.0. Armonk, NY: IBM Corp). Case-control matching was undertaken in Stata version 15.

### Ethical approval

Norwegian Law regulates collection of data by NorCAR.^9^ The use of data and collection of Supplementary data from the patients and next-of-kin is based on written informed consent, and the study was approved by the Regional Ethical Committee; reference number: REK 2016/671.

## Results

Consent to participate was received from 95 (54%) of the survivors eligible for inclusion and available for contact (Fig. 1). Median age was 44 years (35–48), and 26 (27%) were female (Supplemental 2). Compared to non-respondents, respondents were on average four years older, and this was the only significant difference. Among the responders, four could not be included in the analysis of SF-36 data (three delivered incomplete SF-36 questionnaires and one survivor was <18 years at fulfilment of the questionnaire), leaving 91 valid responses to SF-36 (Fig. 1).

The median number of years from OHCA to completion of the questionnaire was 2.1 years (IQR 1.8–2.7). For analysis of data related to exercise-volume, 87 survivors reported their pre-OHCA exercise-volume and could be included in the analysis. Based on reported exercise volume prior to OHCA, 21 (24%) did no regular exercise, and 66 (76%) were regular exercisers (Table 1). Among the regular exercisers, 44 reported 1–4 hours of exercise/week and 22 reported ≥5 hours of exercise/week. There were no significant differences in age, sex, or OHCA event characteristics between those who did no regular exercise and regular exercisers or between the regular exercisers reporting < or ≥5 hours of training. At discharge from hospital, all those who did no regular exercise had a CPC score of 1 (good), compared to three-quarters of the regular exercisers (Table 1). Among the regular exercisers, there was a slightly higher proportion of CPC 1 among those who exercised ≥5 hours/week compared to those who exercised less. OHCA was exercise-related in one-third of the regular exercisers, regardless of exercise volume.

### HRQoL

**Fig. 3:**
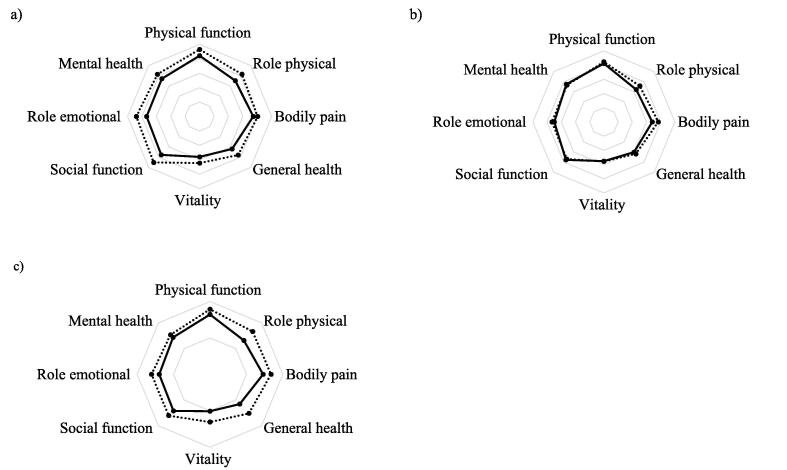
Visual presentation of the between-group differences in SF-36 scores. Comparison of SF-36 scores between groups illustrated with spider plots for; (a) study population (solid line) versus the Norwegian reference population (dotted line), (b) no regular exercise (solid line) versus regular exercisers (dotted line), (c) grouped by <5 hours (solid line) or ≥5 hours (dotted line) of exercise/week prior to OHCA.

Our study participants reported lower SF-36 scores on all scales and both component summary scores compared to controls; mean differences ranged from 6.1 (95% CI −1.4–13.5) for bodily pain and up to 14.8 (95% CI 1.5–15.7) for social function (Fig. 3a, Table 2). Mean differences for physical and mental component scores were 4.9 (95% CI 1.9–8.0) and 5.6 (95% CI 2.5–8.7), respectively, and these were both statistically significant and above the defined minimally important change. The differences for the domains of physical function, general health, and social function were statistically significant and clinically important, while just clinically important for mental health.

Grouped by exercise volume prior to OHCA in the study population, SF-36 scale scores were slightly higher in the regular exercisers compared to those who did no regular exercise, except for social function and mental health. None were statistically significant. (Fig. 3b, Table 3).

Within the regular exercisers, those who reported ≥5 hours of exercise/week had generally higher SF-36 scores compared to those who exercised less (Fig. 3c, Table 3). The group with the highest exercise volume had statistically significant better scores for general health and vitality. The changes were minimally important for bodily pain, general health, vitality, and mental health. The differences in SF-36 component scores were statistically significant and clinically important.

## Discussion

The present study is, as far as we know, the first to compare HRQoL in young survivors of OHCA with an age- and sex-matched reference population, and to assess the impact of exercise volume prior to OHCA on HRQoL. Young Norwegian survivors reported poorer HRQoL assessed by SF-36, including both physical- and mental component scores compared to the reference population. Analysed by whether they reported regular exercise, survivors reported no differences in HRQoL. However, within the group of regular exercisers, OHCA survivors who reported ≥5 hours of exercise/week had higher SF-36 scores indicating better HRQoL than those who exercised less.

As many as 90% of OHCA survivors have a good neurological outcome,^25^ but from qualitative literature, we know that persistent physical and psychological challenges are reported years after cardiac arrest.^6^, ^26^, ^27^ Psychologically, survivors struggle with the contrast between being grateful to be alive and at the same time feel frustrated for the loss of a predictable future.^6^, ^26^ Physical challenges range from objective limitations due to medical restrictions, to limited capacity related to reduced trust and anxiety towards the body after the cardiac arrest.^26^ There may be a disparity between self-assessed quality of life and healthcare personnel’s classification of physical, mental, and cognitive outcomes.^26^ HRQoL is an individual’s perception and can only be expressed by survivors themselves. Self-assessed reports of HRQoL are the quantitative approach to collecting information on the patients’ perspective of health. Since no disease-specific instrument is available for assessing HRQoL after OHCA^4^, ^28^, the COSCA (Core Outcome Set for Cardiac Arrest) initiative recommends using one of the following widely tested generic instruments; the EuroQol EQ-5D-5L, the Health Utility Index version 3 or SF-36, for assessing this population's health and outcomes.^4^, ^29^ An instrument that includes aspects of HRQoL identified as important by survivors of OHCA along with necessary levels of precision is now under development, and will further improve our understanding of mental-, physical and social functioning limitations in this population.^30^ The preliminary name of this disease specific HRQoL tool is the cardiac arrest survivorship and health-related quality of life (CASHQoL) measure.

### HRQoL in young survivors of OHCA compared to an age- and sex-matched reference population

HRQoL is affected by age and sex^5^, which was also the case for our reference population.^11^ Hence, to control for these confounding factors, we compared our population with age- and sex-matched controls. Following this, poorer SF-36 domain scores and component scores were found for survivors of OHCA compared to the reference population. Based on the persistent challenges reported by survivors’ years after the OHCA in qualitative studies,^6^, ^27^ these findings were expected. In contrast to reports from qualitative studies, quantitative data from general population studies suggest that HRQoL among survivors of OHCA are comparable to the general population.^19^, ^20^, ^31^, ^32^, ^33^, ^34^ The majority of these studies present data from adult survivors, mainly without an upper age limit. We included adult survivors with a maximum age of 49 at the time of OHCA.

HRQoL studies of young survivors of OHCA are scarce, but in line with our results, they suggest that young age might be associated with poorer outcomes.^3^, ^6^, ^19^, ^35^, ^36^ There are fewer younger survivors than older survivors due to the epidemiology of cardiac arrest, but their longer lifespan makes them an important subgroup in terms of potential health care use. In general, studies assessing HRQoL among OHCA survivors report good outcomes, and our study confirms the importance of assessing HRQoL for subgroups of survivors. The young survivors in our study had lower scores across all SF-36 scale and component scores. In qualitative studies, young survivors experience difficulties in their return to normal life with work, education, and care for children. They feel that the health services follow-up is tailored towards older patients.^27^ These experiences of mis-match between follow-up and age specific needs, may contribute to the results, and constitutes a target for improvement of guidelines and clinical care. Consideration should be given to adapting follow-up and rehabilitation to both subgroups and individuals, including young survivors of OHCA and their families.^37^

### HRQoL after OHCA related to pre-arrest exercise-volume

As far as we are aware, our study is the first report on HRQoL related to exercise volume prior to OHCA. Within the general population, there have been numerous findings of a positive association between physical activity and HRQoL.^8^, ^38^ For those with very high volume of exercise, data on HRQoL in the general population is limited, but higher levels of HRQoL were reported in United States student-athletes compared to an age- and sex-matched population.^39^ Among survivors of OHCA, an observational study from Australia found better HRQoL after exercise-related OHCA occurring during or ≤1 hour after exercise compared to non-exercise-related OHCA.^40^ The positive association between exercise-related OHCA and HRQoL after OHCA add to the literature of positive associations between physical activity and HRQoL but is not directly comparable to our results.

In the present study, the group who exercised ≥5 hours per week, reported higher scores for the SF-36 scales of vitality and general health as well as physical and mental component summary scores compared to those who exercised less. Better HRQoL after OHCA among those with the highest exercise volume prior to the cardiac arrest could have several explanations. First, better HRQoL in survivors with high exercise volume might be attributed to the positive association between regular physical activity and HRQoL as found in general population studies. Second, there was a higher percentage of survivors with a CPC score worse than one among the regular exercisers who exercised <5 hours per week. The difference was not statistically significant (*p* = 0.10), but we cannot guarantee that it has not affected the results. Our findings of better HRQoL in the group who reported exercising ≥5 hours contribute further to evidence in favour of the benefits associated with exercise, also for OHCA survivors. Surprisingly, there were no significant nor minimally important changes between survivors who did no regular exercise compared to regular exercisers, overall. One explanation might be that in the present study, one-quarter of the regular exercisers had a CPC score worse than one, compared to none of the survivors who reported no regular exercise. Several studies have presented favourable results of HRQoL among survivors with a CPC score of one (=good cerebral performance) compared to CPC of two or worse (=moderate cerebral disability).^41^, ^42^ On the other side, survivors who did no regular exercise had slightly better SF-36 scale scores for social function and mental health, which are also important aspects of HRQoL included in many patient-reported outcome measures.

### Limitations

Data on HRQoL from the reference population was collected in 2002–2003, approximately 15 years prior to our data collection.^15^ Norwegian data suggest that SF-36 values in the Norwegian reference population has been stable during this period.^43^ However, because of a lower response rate in 2015, especially for the youngest participants, it is possible that our comparison to the reference population is affected by this time gap. The response rate among the OHCA survivors was 52% which is comparable with the 56% response rate in the reference population.^15^ However, use of further reminders might have improved the response rate. The proportion of patients with CPC score of one, was equal for respondents and non-respondents. Still, it is possible that the reported HRQoL is affected by a selection bias related to which survivors who chose to participate in the study. Grouped by exercise volume prior to OHCA, those who exercised <5 hours per week had a statistically significantly higher proportion of CPC score worse than one, which might have affected the reported differences for HRQoL between survivors who did and did not exercise regularly. However, CPC score reported in NorCAR is set based on information from the hospital discharge documents and must be interpreted with caution.

The exercise volume prior to OHCA was retrospectively assessed 1–3 years after the OHCA, which might contribute to recall bias.^44^ Reporting exercise volume in three categories may have reduced the bias but assessing hours of exercise/week in pre-defined categories could have compromised the precision level. Unfortunately, data on exercise habits were not collected at the time of the HRQoL assessment to compare exercise volume before and after OHCA.

## Conclusion

This study contributes important evidence on HRQoL in young survivors after OHCA, where existing evidence is scarce. For this small but important subgroup of survivors, lower SF-36 scores compared to the reference population should prompt us to explore how treatment and rehabilitation could be improved and adapted to this age group. Survivors of OHCA reported similar levels of HRQoL independent of whether they exercised on a regular basis prior to OHCA. However, we found an association between high volume of pre-event exercise and higher levels of HRQoL after OHCA, that corresponds well with the existing evidence of beneficial effects of physical activity in general. Further research is recommended in larger populations with more refined instruments for HRQoL and exercise measurement.

## CRediT authorship contribution statement

**Cecilie Benedicte Isern:** Conceptualization, Methodology, Validation, Formal analysis, Investigation, Data curation, Writing – original draft, Writing – review & editing. **Birgitta Blakstad Nilsson:** Conceptualization, Methodology, Validation, Formal analysis, Writing – original draft, Writing – review & editing. **Andrew Garratt:** Conceptualization, Methodology, Validation, Formal analysis, Data curation, Writing – original draft, Writing – review & editing. **Jo Kramer-Johansen:** Conceptualization, Methodology, Validation, Formal analysis, Writing – original draft, Writing – review & editing. **Ingvild B.M. Tjelmeland:** Conceptualization, Methodology, Validation, Formal analysis, Writing – original draft, Writing – review & editing. **Hilde Moseby Berge:** Conceptualization, Methodology, Validation, Formal analysis, Investigation, Writing – original draft, Writing – review & editing.

## Declaration of competing interest

The authors declare the following financial interests/personal relationships which may be considered as potential competing interests: ‘**CBI:** Received un-restricted research grant from Laerdal Foundation for planning and initiation for a Phd-project, which this manuscript constitutes a part of. **BBN:** None to declare. **AMG:** None to declare. **IBMT:** Received unrestricted grant from Laerdal foundation for non-related research project. **JKJ:** Received unrestricted grant from Laerdal foundation for a related research project on HRQoL in all survivors of OHCA. **HMB:** None to declare’.
