# Supplementary material for: Health-related quality of life in young Norwegian survivors of out-of-hospital cardiac arrest related to pre-arrest exercise habits
Source: Resusc Plus. 2023 Oct 5;16:100478. doi: 10.1016/j.resplu.2023.100478 (PMC10560841; doi:10.1016/j.resplu.2023.100478)
Supplement: Supplementary data 1 [file mmc1.docx]

Supplemental 1: Questionnaire designed for the study, translated from Norwegian to English

Questionnaire in the event of cardiac arrest in Norway

| **Category** | **Question** | **Free text / Response alternatives** | **Additional information to responder** |
| --- | --- | --- | --- |
| Information about the patient | Number of the study participant | Free text |  |
|  | Country of birth, patient | Free text |  |
|  | Maternal country of birth | Free text |  |
|  | Father's country of birth | Free text |  |
|  | Skin tone, patient | Black / White / Other | The risk of cardiac arrest is affected by ethnicity. In international studies, ethnicity is described by skin tone, and we therefore ask you to state this |
|  | Maternal skin tone | Black / White / Other |  |
|  | Father's skin tone | Black / White / Other |  |
| Previous medical history | For this category, it is possible to tick multiple answer options per question. | | |
|  | Have you experienced any of the following symptoms at rest/during everyday activity? | Chest pain / Abnormal heavy breathing / Fainting / Near-fainting / Palpitations | Examples of everyday activity: Sleeping, eating, walking to the bus, cutting the lawn |
|  | Have you experienced any of the following symptoms during exercise? | Chest pain / Abnormal heavy breathing / Fainting / Near-fainting / Palpitations |  |
|  | Have you been in contact with a health care provider because of these symptoms? | Yes / No / Do not remember / Have not experienced symptoms prior to cardiac arrest |  |
|  | If so, who were you in contact with? | Free text | (Example: GP xxx, Oslo accident and emergency outpatient clinic, St. Olavs Hospital) |
|  | Have you participated in any kind of cardiac screening before participating in sports? | Yes / No / Do not remember | Screening means that you are offered an examination because you are part of a group and not because you have symptoms. For example, everyone in the club/national team is summoned to a doctor for a cardiac examination |
|  | If so, where and who arranged it? | Free text |  |
|  | If you've attended a cardiac screening, what advice did you get? | Continue as before / Reduce the level of activity / Do not remember |  |
|  | Have you been treated for cardiac disease before? | Yes / No |  |
|  | If yes, - what disease | Free text |  |
|  | What treatment | Free text |  |
| Disease of parents, siblings or children before the age of 50 | Cardiac disease | Tick the box to confirm |  |
|  | If so, in whom and which cardiac disease | Free text |  |
|  | High blood pressure | Tick the box to confirm |  |
|  | If so, in whom | Free text |  |
|  | High cholesterol |  |  |
|  | If so, in whom | Free text |  |
|  | Diabetes | Tick the box to confirm |  |
|  | If so, in whom | Free text |  |
|  | Sudden or unexpected death | Tick the box to confirm |  |
|  | If so, in whom and what was the cause of death | Free text |  |
|  | Do not have information on the medical history of family members | Tick the box to confirm |  |
| Exercise the last year prior to cardiac arrest | How many hours did you exercise in an average week? | 0 hours / <5 hours / 5-10 hours / >10 hours | Exercise is defined as physical effort where you sweat / achieve increased heart rate |
|  | Were you a member of a sports team? | Yes/No | If you didn't exercise, you can skip the rest of the questions in this category |
|  | If so, which team: | Free text |  |
|  | What training/competition level were you at? | Recreational / Competitive athlete / Top athlete |  |
|  | Name a few (max 3) competitions you participated in the last year before your cardiac arrest: | Free text | Example: Sentrumsløpet, local championship in football, Norwegian championship, World championship |
|  | Type of exercise | **Endurance** (example; running, cycling, rowing, cross-country skiing, handball) / **Strength** (example; powerlifting, handball) / **Technical** (example; judo, dance, handball) | You can tick multiple answers. For example, handball, can be strength, endurance and technique depending on the training session |
|  | What sport/activity did you perform?  Main sport/activity: | Free text |  |
|  | Any other sports/activities you participated in regularly? | Free text |  |
|  | How many years have you been practicing current exercise or activity | years |  |
| Did cardiac arrest occur in connection with exercise or physical activity? | During exercise/activity | Tick the box to confirm |  |
|  | If yes, specify the type of exercise or activity | Free text |  |
|  | During competition | Tick the box to confirm |  |
|  | If yes, specify the type of competition | Free text |  |
|  | After exercise/activity/ competition | Tick the box to confirm |  |
|  | If yes, specify how long after? | hours and minutes |  |
| Do you feel that you have been offered sufficient follow-up after the cardiac arrest? | I have received good follow-up from the hospital after discharge | Tick the box to confirm |  |
|  | I have received good follow-up from my general practitioner | Tick the box to confirm |  |
|  | I was offered rehabilitation | Tick the box to confirm |  |
|  | No, I do not feel that I have received sufficient follow-up | Tick the box to confirm |  |
|  | If applicable; I have received follow-up from the sports community | Sports team/ Local sports community / national sports federation / I did not receive follow up from the sports community |  |
| Questions related to the cardiac arrest and details of the resuscitation | Did the cardiac arrest happen at a sports venue/outdoor venue? | Yes/No | Some of the questions may be difficult to answer, if so, choose the response alternative "do not know” |
|  | Specify the location of cardiac arrest | Free text |  |
|  | Was the cardiac arrest witnessed? | Yes / No / Do not know |  |
|  | Who started cardiopulmonary resuscitation? | “Next of kin” or friend / bystander / On-site healthcare professionals / Emergency personnel / Do not know |  |
|  | If the cardiac arrest occurred in a sports arena or fitness center, was there an available defibrillator? | Yes / No / Do not know |  |
|  | Was a defibrillator attached before the arrival of the ambulance / medical doctor? | Yes / No / Do not know |  |
|  | Did the defibrillator deliver a shock before the arrival of the ambulance? | Yes / No / Do not know |  |
|  | Was guidance given to cardiopulmonary resuscitation by those who responded to the emergency telephone line (113)? | Yes / No / Do not know |  |
|  | Field for additional comments | Free text |  |
