# Supplementary material for: Health-related quality of life in young Norwegian survivors of out-of-hospital cardiac arrest related to pre-arrest exercise habits
Source: Resusc Plus. 2023 Oct 5;16:100478. doi: 10.1016/j.resplu.2023.100478 (PMC10560841; doi:10.1016/j.resplu.2023.100478)
Supplement: Supplementary data 2 [file mmc2.docx]

| Supplemental 2: Demographic and event characteristics for young Norwegian survivors of OHCA with presumed cardiac cause, compared between respondents and non-respondents to the study invitation. | | | | | |
| --- | --- | --- | --- | --- | --- |
|  | Respondents | | Non-respondents | |  |
|  | n | % | n | % | P-value |
| Overall | 95 |  | 86 |  |  |
| Age, median (IQR) | 44 (35-48) |  | 40 (26-46) |  | 0.02 |
| Sex, female | 26 | 27 | 21 | 24 | 0.65 |
| OHCA, not witnessed by EMS | 77 |  | 63 |  |  |
| Witnessed by bystander^a^ | 74 | 96 | 56 | 92 | 0.47 |
| Bystander CPR^b^ | 68 | 93 | 51 | 82 | 0.05 |
| First rhythm shockable^†,c^ | 76 | 88 | 59 | 86 | 0.60 |
| CPC-1 ^d^ | 69 | 83 | 56 | 79 | 0.50 |
| Numbers are presented as n and %, except for age.  ^†^The nominator includes cases successfully defibrillated before arrival of EMS, cases that for other reasons had circulation at arrival of EMS are counted as missing.  Missing cases are excluded from the denominators (respondents/non-respondents): ^a^n=0/2, ^b^n=4/1, ^c^n=9/17, ^d^n=12/15. Abbreviations: OHCA; Out-of-hospital Cardiac Arrest, EMS; Emergency Services, CPC; Cerebral Performance Category. | | | | | |
